# Curcumin-Loaded Lactoferrin/Pectin Core–Shell Structured Microgel Nanoparticles: Dual Regulatory Effects in Alleviating Inflammatory Bowel Disease

**DOI:** 10.3390/nu18060921

**Published:** 2026-03-14

**Authors:** Ming-Yu Jin, Sai-Yin Yu, Er-Feng Wang, Henan Zhang, Jing-Yi Xu, Chen Wang, Long-Qing Li, Jing-Kun Yan

**Affiliations:** 1 Dongguan Key Laboratory of Typical Food Precision Design, China National Light Industry Key Laboratory of Healthy Food Development and Nutrition Regulation, School of Life and Health Technology, Dongguan University of Technology, Dongguan 523808, China; 2 School of Pharmacy, Guangdong Pharmaceutical University, Guangzhou 510006, China; 3 Institute of Edible Fungi, Shanghai Academy of Agricultural Sciences, National Engineering Research Center of Edible Fungi, Key Laboratory of Edible Fungi Resources and Utilization (South), Ministry of Agriculture, Shanghai 201403, China; 4 College of Food Science, South China Agricultural University, Guangzhou 510642, China

**Keywords:** inflammatory bowel disease, core–shell microgel nanoparticles, curcumin, dual regulatory effects

## Abstract

Background: Curcumin (Cur) has therapeutic potential for inflammatory bowel disease (IBD) but is limited by its poor bioavailability. Methods: This study demonstrated that Cur-loaded core–shell structured microgel nanoparticles (LF/CP-Cur MN), fabricated through electrostatic complexation between lactoferrin and citrus pectin, followed by Ca^2+^ consolidation, overcome this limitation. Results: These nanoparticles effectively reduced the bitterness and astringency of curcumin while prolonging its release time. In an IBD mouse model, LF/CP-Cur MN treatment mitigated symptoms and inflammation of IBD, and restored intestinal barrier integrity. Crucially, compared with free Cur, the LF/CP-Cur MN enhanced colon-targeted accumulation of Cur and favorably modulated the gut microbiota by increasing beneficial genera like *Lactobacillus* and *Dubosiella*, while suppressing harmful genera like *Enterobacter*, thereby promoting levels of acetate, propionate, and butyrate. Conclusions: These findings highlight the potential of the LF/CP-Cur MN to improve Cur bioaccessibility and exert dual functional roles in modulating gut microbiota and alleviating inflammation, thus offering a promising dietary strategy for the management of IBD.

## 1. Introduction

Inflammatory bowel disease (IBD) is a chronic nonspecific intestinal inflammation of unknown etiology, characterized by destructive and relapsing tissue pathology, including dysregulated local immune responses and impaired intestinal barrier function [1]. The incidence of IBD has risen sharply in recent years, posing significant threats to public health due to its recurrent nature and challenges in achieving remission [2]. According to the U.S. Optum Research Database (2007–2016), IBD patients bear an annual direct and indirect financial burden of $15,000 [3]. Current therapies primarily rely on anti-inflammatory drugs, immunosuppressants, and biologics [4]. However, these interventions frequently trigger a wide array of adverse effects such as headaches, nausea, osteoporosis, digestive disorders, mood disturbances, and cardio-vascular complications [5,6]. Therefore, the development of novel therapeutic targets and strategies for IBD is of critical importance for its scientific prevention and management.

Extensive studies have demonstrated that polyphenols derived from dietary and plant-based sources can effectively prevent and ameliorate IBD-associated colonic damage and immune dysregulation, positioning them as safe and potent adjuncts for dietary intervention strategies [7,8]. Curcumin (Cur), a natural polyphenolic compound derived from turmeric (*Curcuma longa* L.), has attracted considerable attention for its anti-inflammatory and antioxidant properties [9]. Mechanistic studies have demonstrated that Cur can target Toll-like receptors, abrogate the NF-κB signaling pathway, and inhibit the MAPK and JAK/STAT pathways, exhibiting notable efficacy, safety, patient tolerability, and cost-effectiveness [9,10,11]. However, Cur possesses poor aqueous solubility (only 0.4 mg/mL at pH 7.3), and it undergoes rapid metabolic clearance in vivo through reduction and conjugation reactions, resulting in low bioavailability [9,11]. These limitations significantly impede its clinical development and application as a therapeutic agent. Consequently, enhancing the bioavailability and stability of oral Cur to achieve targeted delivery to the colon and controlled release has emerged as a pivotal challenge in advancing dietary interventions for IBD.

Recent studies have shown that Cur engineered through nanotechnology serves as an efficient drug delivery platform, enabling precise targeting of tissues in the human body. For instance, Liang et al. developed a poly(diselenide-oxalate-Cur) nanoparticle (SeOC-NP) with dual-reactive oxygen species (ROS) sensitive chemical moieties (diselenide and peroxalate ester bonds). The SeOC-NP significantly inhibited the oxidative degradation of Cur and exerted an efficient inflammatory treatment [12]. In addition, Jin et al. prepared a Cur@Fe&TA nanodrug delivery system by constructing an Fe^3+^/tannic acid (TA) metal-polyphenol network with encapsulated Cur. The Cur@Fe&TA nanodrug exhibited good stability, drug release behavior, and biocompatibility, and improved gastrointestinal cytopathology in an IBD mouse model [13]. What’s more, CD44 and GM dual-targeted nanoparticles loaded with CUR (CUR@Chs-PNC NPs) were derived from a quaternized chitosan and surface functionalization with chondroitin sulfate [14]. CUR@Chs-PNC NPs reduced the disease activity index in IBD mice, alleviated oxidative stress and inflammatory conditions, promoted the production of short-chain fatty acids (SCFAs), modulated immune cells, and maintained gut microbiome homeostasis. Although nanoparticle-based delivery systems can improve the bioavailability of Cur, conventional methods involve chemical modifications that raise safety concerns, and the functionality of the carrier materials remains underutilized. Therefore, it is imperative to explore novel nanoparticles that can safely and efficiently deliver Cur while achieving synergistic effects through carrier functionality.

To address these issues, we initially employed lactoferrin (LF) and citrus pectin (CP) as safe and effective core–shell carriers through electrostatic self-assembly, which significantly prolonged the in vitro release of Cur [15]. However, the anti-inflammatory mechanism of this system remained unclear. In this study, we developed an oral colon-targeting delivery system based on our earlier work, in which Ca^2+^ and CP were used to stabilize the LF/CP core–shell structure and encapsulate Cur (LF/CP-Cur MN). We further evaluated the ability of this system to modulate the gut microbiota and attenuate colitis in a dextran sulfate sodium (DSS)-induced mouse model. This work provides new insights for the design of oral drug delivery carriers.

## 2. Materials and Methods

### 2.1. Materials and Reagents

CP, LF (80 kDa) and Cur were sourced from Shanghai Yuanye Biotechnology Co., Ltd. (Shanghai, China). Dextran sulfate sodium salt (DSS, 36–50 kDa) was procured from MP Biomedicals (Santa Ana, CA, USA). Hematoxylin-eosin (H&E) and alcian blue (AB) staining kits were obtained from Nanjing Jiancheng Bioengineering Institute (Nanjing, China). Molecular biology reagents, including Total DNA/RNA extraction kits, first-strand cDNA synthesis kit, polymerase chain reaction (PCR) master mix, and sequence-specific primers, were supplied by TianGen Biotech (Beijing, China). Mouse-specific enzyme-linked immunosorbent assay (ELISA) kits for tumor necrosis factor (TNF)-α, interleukin (IL)-1β, and IL-6 quantification were purchased from Shanghai Jianglai Biotechnology Co., Ltd. (Shanghai, China).

### 2.2. LF/CP-Cur MN Preparation

**Figure 1:**
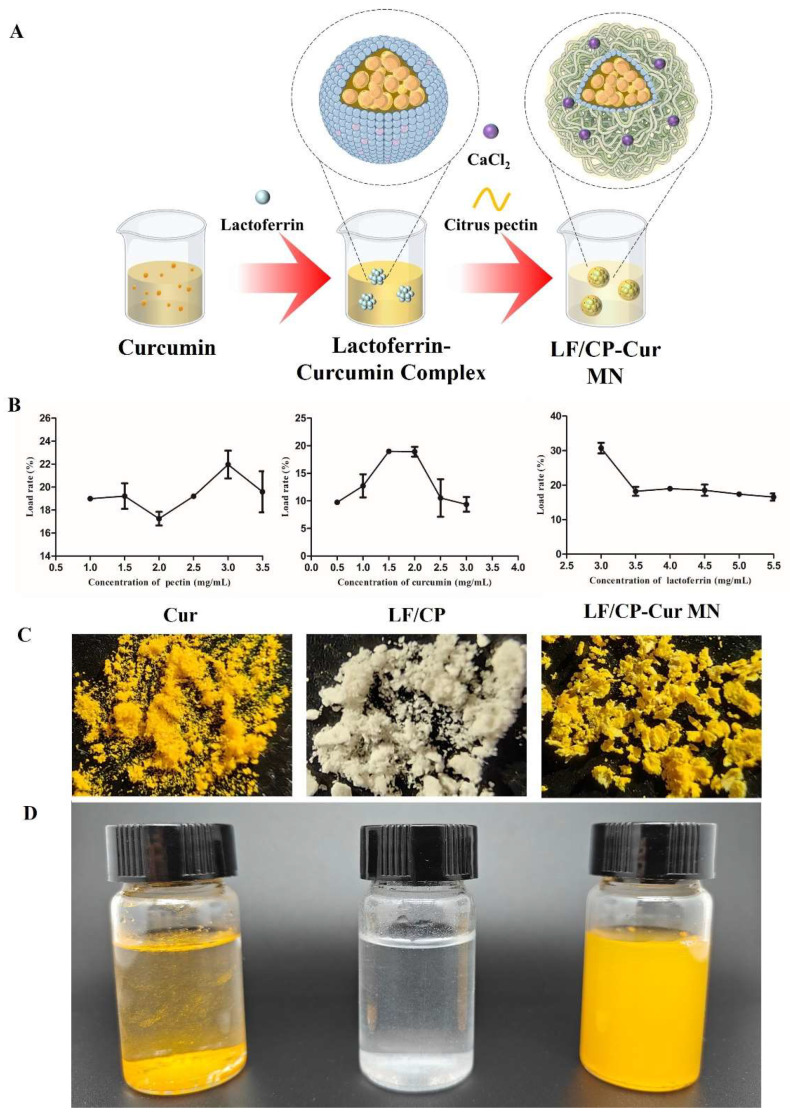
Fabrication and physical property of LF/CP-Cur MN. (**A**) Schematic illustration of the LF/CP-Cur MN, (**B**) Univariate optimization of curcumin loading efficiency in LF/CP-Cur MN, (**C**) Visual appearance and (**D**) SEM images showing the surface morphology of Cur, LF/CP, and LF/CP-Cur MN. Data are reported as means ± SD (*n* = 3).

The method of LF/CP-Cur MN preparation makes some modifications according to Yan’s operation [15]. LF and CP powders dissolved in deionized water, respectively, and Cur dissolved in absolute ethyl alcohol (Figure 1A). The LF solution was mixed with an equal volume of Cur solution, stirred at 25 °C for 20 min, and subsequently heat-denatured at 90 °C for 30 min. Unbound Cur was removed via centrifugation (3000 rpm, 10 min) to obtain LF-Cur complexes. The resultant LF-Cur complexes were mixed with CP solution at a 1:3 (*v*/*v*) ratio and stirred for 60 min, followed by crosslinking with 0.5 M CaCl_2_ for 20 min. The resulting complexes (LF/CP-Cur MN) were collected by centrifugation at 4000 rpm for 10 min and then lyophilized for subsequent use.

### 2.3. Single-Factor Optimization Experiment of LF/CP-Cur MN

Single-factor optimization of LF/CP-Cur MN was conducted by evaluating the effects of varying concentrations of Cur (0.5–3.0 mg/mL), LF (3.0–5.5 mg/mL), and CP (1.0–3.5 mg/mL). During each test, the other two components were held constant at fixed levels: CP (1.0 mg/mL), LF (4.0 mg/mL) and Cur (1.5 mg/mL), respectively. Following this protocol, the loading capacity (LC) of Cur was quantified using IsoPlane SCT 320 UV-Vis spectroscopy (Princeton Instruments, Inc., Trenton, NJ, USA) at 427 nm. Cur concentration was quantified by comparison with a standard curve of free Cur (R^2^ = 0.9984). All measurements were conducted in triplicate at 25 °C. The LC (%) was calculated as:

$$LC\left(\%\right)\;=\;\left(m_{Cur\;in\;LF/CP-Cur\;MN\;}/m_{LF/CP-Cur\;MN}\right)\times 100$$

where *m*_Cur in LF/CP-Cur MN_ represents the mass of Cur encapsulated in the nanoparticles (μg), and *m*_LF/CP-Cur MN_ denotes the total mass of the LF/CP-Cur MN (μg). These results revealed a concentration-dependent modulation of LC, thereby identifying optimal parameters for maximizing encapsulation efficiency.

### 2.4. Dynamic Light Scattering (DLS) Analysis

The zeta potential and particle size of both LF/CP core–shell and LF/CP-Cur MN formulations were quantitatively assessed using a Zetasizer Nano ZS90 system (Malvern Panalytical, Malvern, UK) [15].

### 2.5. Scanning Electron Microscopy (SEM) Analysis

A small amount of each freeze-dried sample (Cur, LF/CP, and LF/CP-Cur MN) was mounted on aluminum stubs, sputter-coated with gold for 60 s. Subsequently, the prepared samples were analyzed using a COXEM EM-30 Plus scanning electron microscope (Korea Cusam Co., Ltd., Daejeon, Republic of Korea) under high vacuum conditions to observe surface morphology.

### 2.6. X-Ray Diffraction (XRD) Analysis

The crystalline and amorphous properties of Cur, LF/CP, and LF/CP-Cur MN solid samples were characterized by XRD using a Dandong Haoyuan DX-2800 diffractometer equipped with a Cu Kα radiation source (*λ* = 0.15406 nm). XRD patterns were acquired over a 2θ range of 5–50° under operational conditions of 40 kV and 40 mA, with a scanning rate of 4°·min^−1^, a step size of 0.02°, and a dwell time of 0.2 s per step [15]. The X-ray tube was operated at 30 kV and 20 mA to optimize detection sensitivity.

### 2.7. Fourier Transform Infrared Spectroscopy (FT-IR) Analysis

FT-IR spectra of LF, CP, free Cur, LF/CP, and LF/CP-Cur MN samples were recorded using a Thermo Fisher Scientific Nicolet Nexus 670 FTIR spectrometer (Madison, WI, USA) in the wavenumber range of 500–4000 cm^−1^ via the potassium bromide (KBr) pellet method. All measurements were performed under identical instrumental conditions with background correction relative to air.

### 2.8. Thermal Stability Analysis

Differential scanning calorimetry (DSC) thermograms of free Cur, LF/CP, and LF/CP-Cur MN were acquired using a Mettler Toledo DSC 822e thermal analyzer (PerkinElmer, USA). Approximately 3.0 mg of each sample was placed into aluminum crucibles and hermetically sealed with aluminum lids. Thermal analysis was performed under a dry nitrogen atmosphere with a flow rate of 20 mL/min, using a heating rate of 10 °C/min over a temperature range of 25–300 °C. An empty sealed crucible was used as the reference baseline for background subtraction.

### 2.9. Taste Profile Analysis by Electronic Tongue

To ensure consistent curcumin (Cur) content, the test samples were prepared by dissolving 3.3 mg/mL Cur and 10.0 mg/mL LF/CP-Cur MN, in an aqueous solution containing 1% Tween-80. The analysis was performed using an electronic tongue system (SA402B Controller, Insent, Kanagawa, Japan). The signal acquisition time for each sample was 120 s, with a sampling rate of 1 Hz. The system is equipped with five sensors for sourness, saltiness, umami, bitterness, and richness (or astringency). The average value of the stable signal from 100 to 120 s was used for subsequent data analysis.

### 2.10. In Vitro Drug Release Studies

The method of in vitro release of Cur from LF/CP-Cur MN makes some modifications according to Yan’s operation [15]. The LF/CP-Cur MN was evaluated in buffer solutions at pH 1.5 (HCl), 4.5 (HCl), and 7.4 (PBS), each supplemented with 0.5% (*v*/*v*) Tween-80. A dispersion was prepared by suspending 20 mg of LF/CP-Cur MN in 20 mL PBS. Then, 2 mL of this dispersion was placed in a dialysis bag with a molecular weight cutoff (MWCO) of 8000–14,000 Da. The dialysis bag was immersed in 50 mL of the corresponding buffer solution and incubated at 37 °C with shaking at 120 rpm. Samples of 1 mL were collected at regular intervals (0–120 h) and replaced with fresh buffer solution. The concentration of Cur was determined using UV-Vis spectroscopy at 427 nm absorbance.

### 2.11. Animals and Diets

A total of 32 male C57BL/6 mice (6 weeks old, 20–24 g) were purchased from specific pathogen-free Biotechnology Co., Ltd. (Beijing, China) and housed under controlled conditions (22 °C, 55% humidity, 12-h light/dark cycle). The experiments were conducted in strict accordance with the protocol approved by South China Agricultural University (SYXK[Guangdong]2024-0136). After a one-week acclimatization period, 32 mice were randomly divided into four groups (*n* = 8; 2 cages, 4 mice/cage). The groups included a control group (CON) receiving normal drinking water, an IBD group (DSS) induced with 2% DSS water (DSS), a 2% DSS water + 100 mg/kg/d Cur intervention group (DSS + Cur) [11], and a 2% DSS water + 330 mg/kg/d LF/CP-Cur MN intervention group (DSS + LF/CP-Cur MN). A uniform dose of Cur was maintained in the intervention groups. DSS was administered over a period of 7 days [16], while Cur and LF/CP-Cur MN interventions were conducted over 11 days, respectively. All mice had free access to food and water. Body weight, stool consistency and the presence of fecal occult/gross blood were monitored daily, with the frequency increased to twice daily during the peak phase of colitis (Days 5–7). To minimize pain and distress, all animals were handled gently and housed in a quiet environment. Animals exhibiting severe clinical symptoms (e.g., extreme lethargy, kyphosis, inability to ingest food or water, or a moribund state) or manifesting clear signs of distress (e.g., hypoactivity, piloerection, and social isolation) will be humanely euthanized immediately to terminate further suffering. Euthanasia at endpoint (Day 11) followed AVMA Guidelines for the Euthanasia of Animals: 2020 Edition via stepwise CO_2_ inhalation. Animals were placed in a ventilated chamber pre-filled with 30% CO_2_ (1 L/min, 1 min), then exposed to 100% CO_2_ at 20% chamber volume/min (fill time ≤ 3 min). Cervical dislocation was performed within 60 s after respiratory arrest. Death was confirmed by absence of corneal reflex, pupillary light response, and spontaneous breathing for ≥5 min.

### 2.12. Measurements of Body Weight, Colon Length, and Disease Activity Index (DAI)

The longitudinal monitoring included daily assessment of body weight fluctuations (expressed as percentage change from baseline) and fecal consistency scoring (0–4 scale: formed stool to liquid diarrhea). Post-euthanasia, colonic specimens were immediately measured using digital calipers alongside a reference scale. DAI was calculated according to established criteria [17], incorporating three parameters: Weight loss: 0 (none) to 4 (>20% reduction), Stool consistency: 0 (normal) to 4 (watery diarrhea), Rectal bleeding: 0 (absent) to 4 (gross bleeding). Final DAI = (Weight score + Stool score + Bleeding score)/3.

### 2.13. Histopathology and Mucus Analysis

Colonic tissues were fixed in 4% paraformaldehyde (>24 h), paraffin-embedded, and sectioned at 5 μm thickness. Following sequential dewaxing (xylene → 100–0% ethanol gradient) and staining with H&E or AB using commercial kits (protocol-optimized) from Nanjing Jiancheng Bioengineering Institute (Nanjing, China), sections were dehydrated through ascending ethanol series (50–100%) and xylene. After dehydration, pathological sections were mounted with neutral balsam. Histomorphological analysis was performed using an Axio Observer A1 microscope (Zeiss, Oberkochen, Germany) at a magnification of 20×.

### 2.14. Analysis of Cur Content in Colon

The mouse colon was weighed, minced, and extracted with 500 μL of 50% ethanol via vortex mixing for 1 h. After centrifugation (4000 rpm, 20 min), the supernatant was carefully collected. Absorbance was measured at 432 nm to construct a standard curve for the quantification of Cur content in the colon.

### 2.15. Cytokine Quantification by Enzyme-Linked Immunoassay (ELISA)

The blood samples were allowed to clot at 37 °C for 30 min, followed by centrifugation (5000 rpm, 10 min), after which the serum was collected. Serum concentrations of inflammatory cytokines, including TNF-*α*, IL-1*β* and IL-6, were measured using commercially available ELISA kits (Shanghai Jianglai Biotechnology Co., Ltd., Shanghai, China) in accordance with the manufacturer’s protocols.

### 2.16. 16s rRNA Gene Profiles

Fecal samples (0.25–0.50 g or 200 μL liquid) were suspended in lysis buffer (500 μL SA + 100 μL SC) containing 0.25 g of grinding beads and subjected to vortex mixing, cold homogenization, and heat lysis at 70 °C for 15 min. The resulting mixture was centrifuged to collect the supernatant. Target regions were amplified using standard primers, including bacterial 16S (V3-V4/V4-V5/V4), endophytic 16S V3-V4, fungal ITS1/ITS2, archaeal 16S V3-V4, and eukaryotic 18S V4/V7 using standard primers (e.g., 338F/806R for 16S V3-V4). PCR products were verified via 2% agarose gel electrophoresis, purified using AMPure XP beads (Beckman Coulter, Brea, CA, USA), and sequenced on the Illumina MiSeq platform with a 2 × 300 bp paired-end read configuration.

### 2.17. Determination of Short-Chain Fatty Acids (SCFAs)

A 0.2 g aliquot of cecal content was homogenized in 1 mL sterile water for 30 s, followed by centrifugation at 10,000 rpm for 5 min. The resulting supernatant (1.0 mL) was acidified with 0.1 mL of 50% H_2_SO_4_, then extracted with 1.0 mL of diethyl ether, mixed, and centrifuged (10,000 rpm, 5 min). After incubation at 4 °C for 3 h, the diethyl ether layer was collected and filtered (0.22 μm) for GC analysis.

GC conditions: Analysis was performed on an Agilent 6890N GC system equipped with an FID detector (Agilent, Santa Clara, CA, USA). Chromatographic separation was achieved on a polar FFAP elastic quartz capillary column (30 m × 0.25 mm × 0.25 μm) with the following temperature program: initial temperature held at 100 °C for 1 min. This was followed by a ramp of 5° C/min to 150 °C, where it was held for 5 min. High-purity nitrogen (≥99.999%) was used as the carrier gas at a constant flow rate of 2 mL/min. The injector and detector temperatures were maintained at 250 °C and 300 °C, respectively. A 1.0 μL sample was injected in split mode.

### 2.18. Statistical Analysis

All data are presented as mean ± SEM. Statistical analyses were conducted using SPSS 17.0, and graphical representations were generated using GraphPad Prism 5 and Microsoft Office 2016. Significant differences were assessed by one-way ANOVA with Tukey’s post hoc test (* *p* < 0.05, ** *p* < 0.01, *** *p* < 0.001).

## 3. Results

### 3.1. Optimized LC and Dissolved State of LF/CP-Cur MN

The LF is a single-chain glycoprotein with a molecular weight of approximately 80 kDa, consisting of approximately 700 amino acid residues. It is structurally organized into two homologous lobes—the *N*-terminal lobe (N-lobe) and the *C*-terminal lobe (C-lobe)—each harboring an iron-binding cavity [18]. The N-lobe and C-lobe cavities (natural iron-binding sites) of LF can encapsulate the hydrophobic aromatic rings of Cur through hydrophobic interactions, thereby forming stable host-guest complexes. Our previous research designed core–shell structured LF/pectin PEC NPs through electrostatic complexation between positively charged thermally denatured LF and negatively charged anionic pectin [19]. This nanoparticle effectively reduces the hydrophobic effect of Cur and prolongs the release time of Cur. In this study, the process was modified to maximize the Cur LC of LF: lactoferrin was first complexed with curcumin and then heat-denatured, reversing the original order. Furthermore, the core–shell stability of the LF/CP-Cur MN system was enhanced by leveraging the specific Ca^2+^ coordination with CP that drives the ‘egg-box’ model complexation [20].

A single-factor analysis was conducted with the Cur concentration gradient (0.5–3.0 mg/mL) as the variable. The results shown in Figure 1B demonstrate that, under equal volume conditions, the complex formed by 4 mg/mL LF and 1.5 mg/mL Cur was the most abundant, with a LC of 18.99 ± 0.09%. Moreover, the Cur loading capacities in LF/CP-Cur MN at Cur concentration of 1.5 mg/mL (18.99 ± 0.09%) and 2.0 mg/mL (18.92 ± 1.25%), along with the CP concentration, were maintained at 1 mg/mL throughout the process. When further exploring LF concentration gradients (3.0–5.5 mg/mL) in LF/CP-Cur MN prepared with 1 mg/mL CP and 1.5 mg/mL Cur, a LF concentration of 3.0 mg/mL resulted in the highest Cur loading efficiency (30.74 ± 2.18%).

The amino groups in LF are coupled with reactive functional groups such as carboxyl groups in CP through electrostatic interactions [21]. A higher Cur LC (21.97 ± 1.71%) was achieved at a CP concentration of 3.0 mg/mL. This was tested with Cur and LF concentrations fixed at 1.5 mg/mL and 4 mg/mL, respectively, across a CP concentration gradient ranging from 1.0 to 3.5 mg/mL. Although high CP concentrations enhance encapsulation efficiency, they also increase the specific gravity of the core–shell LF/CP composite, thereby reducing Cur loading efficiency. Based on comprehensive single-factor analysis, the optimized LF/CP-Cur MN formulation (1 mg/mL CP, 1.5 mg/mL Cur, and 3.0 mg/mL LF) achieved the highest Cur loading efficiency—significantly surpassing previously reported values of ~13.4% [15]. Maria et al. [22] reported an encapsulation efficiency of ~0.089% for whey protein isolate-chitosan (WPI-CH) emulsion gels used in Cur encapsulation, which is lower than the encapsulation efficiency observed for the LF/CP-Cur MN in this study.

The aforementioned results indicate that the LF/CP-Cur MN, prepared under conditions of 1 mg/mL CP, 1.5 mg/mL Cur, and 3.0 mg/mL LF, achieves the highest Cur loading efficiency (30.74%). This value is markedly higher than the embedding rate (~13.4%) previously reported [15]. Furthermore, the particle size of the LF/CP-Cur MN solution was measured using a laser particle size analyzer to be approximately 208 nm, with a Zeta potential of around −32.1 mV.

As shown in Figure 1C,D, the physical state of Cur powder and its aqueous solution at room temperature is presented. Both the core–shell LF/CP and LF/CP-Cur MN formed homogeneous aqueous dispersion following 3 h of mixing at room temperature. The yellow coloration observed in the LF/CP-Cur MN dispersion was attributed to the presence of Cur, indicating successful encapsulation within the core–shell LF/CP structure. This encapsulation effectively shielded the hydrophobic domains of Cur, thus enhancing its water solubility [21].

### 3.2. Structural Characterization and Surface Morphology of LF/CP-Cur MN

**Figure 2:**
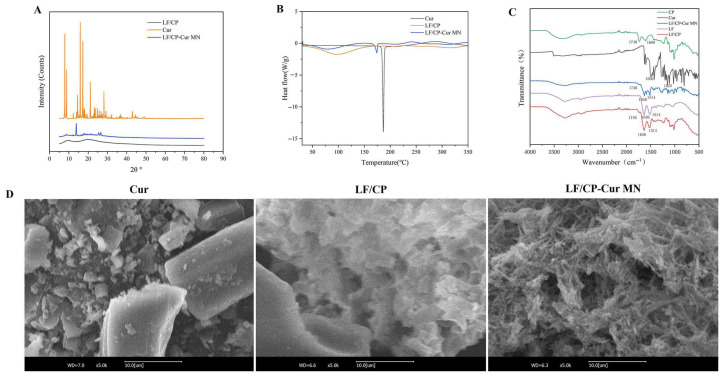
Structure characteristics of LF/CP-Cur MN. (**A**) XRD, (**B**) DSC, (**C**) FT-IR, (**D**) SEM.

The XRD patterns presented in Figure 2A illustrate the crystalline structures of free Cur, LF/CP, and LF/CP-Cur MN. The results show that Cur exhibits multiple characteristic diffraction peaks within the 2θ range of 5° to 50°, with prominent peaks observed near 7.9°, 8.8°, 14.5°, 15.9°, 17.2°, and 28.1°, respectively, which are indicative of its crystalline nature [15]. In contrast, the core–shell LF/CP displays a smooth diffraction pattern devoid of distinct peaks, indicating an amorphous structure. For LF/CP-Cur MN, characteristic diffraction peaks are observed at 13.8°, 25.5°, and 26.6°, indicating that Cur undergoes a transition from a crystalline to an amorphous state after encapsulation. This suggests that Cur is molecularly dispersed within the LF/CP matrix. The amorphous state of Cur is known to enhance its absorption and bioavailability in the human body. Furthermore, no crystalline peaks are detected in the core–shell LF/CP material. These observations are consistent with previous reports on Cur encapsulated in starch nanocapsules, which also exhibited amorphous characteristics [23].

The DSC results revealed that the thermal analysis curve of free Cur exhibited a pronounced endothermic peak at approximately 186.67 °C (Figure 2B), corresponding to the melting point of Cur crystals, thereby further confirming its highly crystalline nature [15]. However, a small endothermic peak corresponding to Cur was observed at 174 °C in the LF/CP-Cur MN, indicating a substantial reduction in the crystallinity of Cur, which is consistent with the XRD analysis (Figure 2A). This observation further demonstrates the successful encapsulation of Cur within the LF/CP microgel nanoparticles, resulting in the formation of a high-energy amorphous state.

The FT-IR analysis of LF/CP-Cur MN clarified the principal chemical interactions underlying nanoparticle formation (Figure 2C). In the CP spectrum, the absorption peak observed near 1015 cm^−1^ can be attributed to the stretching and bending vibrations of C–O bonds within the pyranose rings of carbohydrates [24]. The peak at 1740 cm^−1^ corresponds to the stretching vibrations of methyl ester groups (COOCH_3_) and undissociated carboxylic acid groups (COOH) [15]. The peak around 1606 cm^−1^ is assigned to the asymmetric stretching vibration of carboxylate anions (COO^−^). The LF spectrum showed characteristic amide I (1636 cm^−1^, C=O stretching vibration) and amide II (1517 cm^−1^, N-H bending and C-N stretching vibrations) protein bands [15]. The LF/CP spectrum preserved characteristic features from both components, yet demonstrated the absence of the 1606 cm^−1^ peak, indicating an interaction between CP (carboxylate groups) and LF (amino groups) or Ca^2+^. Furthermore, Cur exhibited characteristic absorption peaks at 3505 cm^−1^ (phenolic O-H stretching), 1025 cm^−1^ (C-O-C vibration), and 1260 cm^−1^ (C=O stretching), which are consistent with its structural characteristics [25]. Notably, the spectrum showed well-preserved characteristic peaks at around 1744 cm^−1^, 1636 cm^−1^, 1514 cm^−1^, 1260 cm^−1^, and 1025 cm^−1^, corresponding to CP, LF, and LF/CP vibrational modes. These peals are consistent with the composite nature of the nanoparticles. The absence of the characteristic Cur O-H stretching vibration band at 3505 cm^−1^ and the CP COO^−^ asymmetric stretching peak at 1740 cm^−1^ in the LF/CP-Cur MN spectrum suggests successful encapsulation of Cur within the nanoparticles.

SEM was employed to observe the surface morphology of the samples (Figure 2D). It was found that the Cur particles exhibited a smooth and finely fragmented structure, while the LF/CP surface displayed a flocculent, smooth, loose, and porous morphology. Furthermore, the LF/CP-Cur MN displayed a well-defined fibrous network with an alternately porous architecture and an absence of coarse particles on the surface, indicating successful encapsulation of Cur within the core–shell LF/CP matrix.

### 3.3. In Vitro Sensory Evaluation and Drug Release Analysis of LF/CP-Cur MN

**Figure 3:**
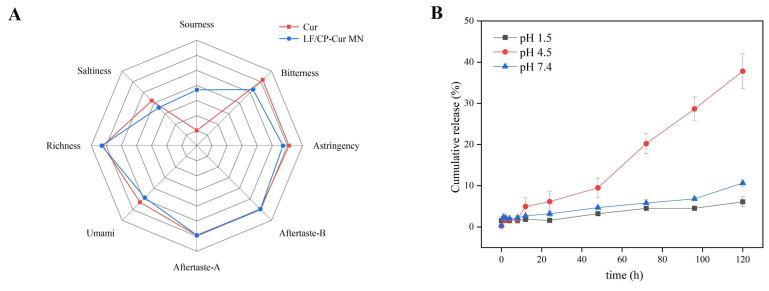
(**A**) Sensory evaluation of LF/CP-Cur MN and (**B**) in vitro drug release analysis of LF/CP-Cur MN at pH 1.5, 4.5 and 7.4.

The bitter and astringent taste of Cur significantly compromises its palatability. To validate this hypothesis, a comprehensive and objective evaluation of the sensory profiles of free Cur and LF/CP-Cur MN was conducted using an electronic tongue (Figure 3A). The data revealed that the core–shell LF/CP encapsulation substantially mitigated the bitterness and astringency of Cur. Concurrently, a noticeable increase in sourness was detected, which can be attributed to the CP within the shell layer. In summary, LF/CP-Cur MN effectively improved the oral sensory attributes of Cur by masking its undesirable taste characteristics.

To further investigate the delivery characteristics of LF/CP-Cur MN, its release profile was evaluated under simulated gastrointestinal pH conditions. The results demonstrated a pronounced pH-responsive release behavior (Figure 3B). In a highly acidic environment (pH 1.5, simulating gastric fluid), LF/CP-Cur MN exhibited remarkable stability. Its cumulative release rates of merely 1.59% at 24 h and 6.08% at 120 h, indicating effective protection of Cur against degradation and premature release in the stomach (Figure 3B). Under weakly acidic conditions (pH 4.5), the release rate increased progressively, with cumulative releases reaching 1.91% at 8 h and 37.80% at 120 h. In a neutral environment (pH 7.4, simulating intestinal fluid), the cumulative release reached 2.69% at 12 h and increased to 10.63% at 120 h. Compared to the previously prepared LF/CP PEC NPs, LF/CP-Cur MN significantly prolonged the release time of Cur. Within 120 h, the cumulative release rates of Cur from LF/CP PEC NPs were 78.36% and 67.18% at pH 4.5 and 7.4, respectively [15]; whereas LF/CP-Cur MN demonstrated a significantly slower release profile throughout the entire period. This can be attributed to the formation of a complex between Ca^2+^ and CP, which stabilized the core–shell structure. It was demonstrated that the cumulative release of CUR from CUR@Chs-PNC NPs was approximately 17% after 2 h in a simulated gastric environment [14]. Following an additional 4 h of incubation in a simulated intestinal environment, the cumulative release reached 36%. Based on the reported gastrointestinal transit times (approximately 4 h for gastric emptying and 6 h for small intestinal transit) [26], the estimated cumulative release of Cur following these critical phases was approximately 3.47%. This suggests that the majority of Cur remains stably encapsulated within the LF/CP-Cur MN until reaching the colon, where it is then released to exert its localized therapeutic effects.

### 3.4. Amelioration of Clinical Symptoms in IBD Mice by LF/CP-Cur MN

**Figure 4:**
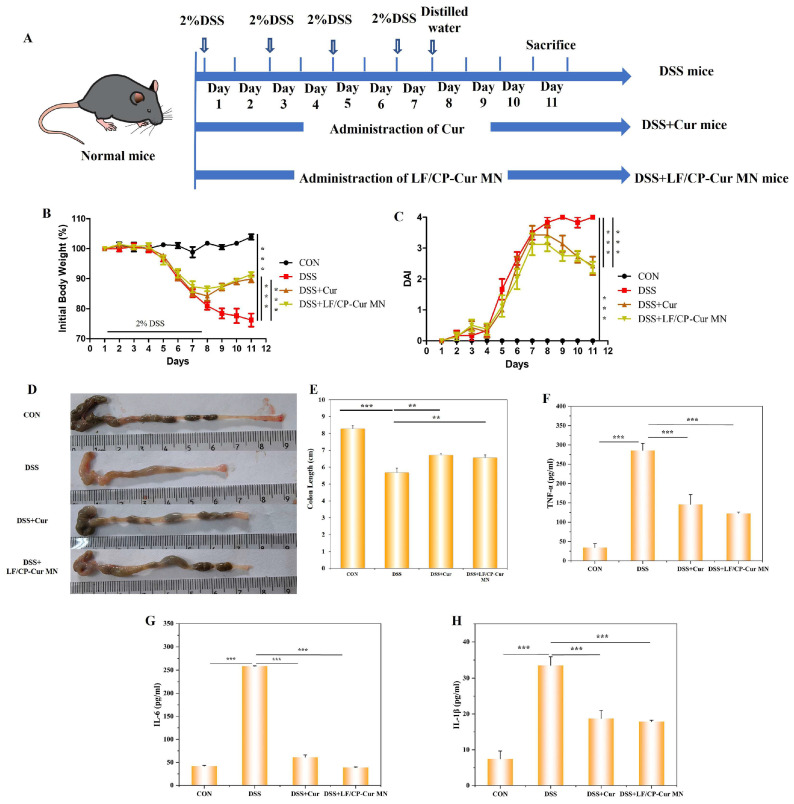
Effect of LF/CP-Cur MN administration on the clinical symptoms of IBD mice during the study period. (**A**) Body weight changes, (**B**) disease activity index (DAI) scores during the experiment, (**C**) representative images of colons, (**D**) representative image of the colon, (**E**) colon lengths, the content of (**F**) TNF-α, (**G**) IL-6, (**H**) IL-1β in serum. Data are shown as mean ± SEM (*n* = 3); ** *p* < 0.01, *** *p* < 0.001.

Clinical trials have shown that Cur effectively alleviates symptoms in patients with IBD [27]. However, its therapeutic potential is limited by low oral bioavailability, which results from poor water solubility, chemical instability, extensive intestinal metabolism, and rapid systemic clearance. This study investigated the anti-inflammatory effects of LF/CP-Cur MN using a DSS-induced IBD mouse model (Figure 4A). The results showed that the DSS-induced IBD mice exhibited a progressive decline in body weight starting on day 4, accompanied by the onset of loose stools and, subsequently, bloody feces from day 5 onward (Figure 4B). The differences compared to the CON group were statistically significant (*p* < 0.001), indicating successful establishment of the IBD mouse model. Cur intervention effectively mitigated weight loss in IBD mice starting from the eighth day, with a comparable therapeutic effect observed in mice treated with LF/CP-Cur MN. The DAI, calculated based on daily body weight changes and stool consistency, showed that DAI values in IBD mice continued to increase after day 7. In contrast, mice treated with free Cur and LF/CP-Cur MN exhibited a declining trend. Significant differences were observed among the DSS + Cur group, the DSS + LF/CP-Cur MN group, and the DSS group (*p* < 0.001) (Figure 4C).

In the DSS group, a small amount of watery stool was observed in the colons of mice, accompanied by colonic tissue edema and significant shortening attributable to inflammation (5.67 ± 0.28 cm), which was markedly shorter than that in the CON group (8.27 ± 0.19 cm) (Figure 4D,E). The difference between the two groups was statistically significant (*p* < 0.05). After intervention with free Cur and LF/CP-Cur MN, the colon length increased by 18.23% and 15.59%, respectively, compared to the DSS group. No statistically significant difference was observed between the two treatment groups (*p* > 0.05), indicating that LF/CP-Cur MN and free Cur exert comparable therapeutic effects in alleviating colonic inflammation in IBD mice.

Pro-inflammatory cytokines, such as IL-1*β*, TNF-*α*, and IL-6, are key mediators secreted during the inflammatory response [28]. This study evaluated the effect of LF/CP-Cur MN in suppressing these inflammatory cytokines. As illustrated in Figure 4F–H, free Cur administration significantly reduced serum levels of TNF-*α*, IL-6, and IL-1*β* in IBD mice by 48.94%, 76.32%, and 44.04%, respectively (*p* < 0.05). Meanwhile, treatment with LF/CP-Cur MN achieved comparable reductions of 57.19%, 84.95%, and 46.58%, respectively (*p* < 0.05), demonstrating its potent anti-inflammatory potential. These results indicate that the core anti-inflammatory efficacy of Cur was effectively maintained following its encapsulation within nanoparticles. This demonstrates that LF/CP-Cur MN is comparable to free Cur in alleviating inflammatory responses.

### 3.5. Protective Effect of LF/CP-Cur MN on the Intestinal Mucosal Barrier and Enhanced Colonic Accumulation of Cur in IBD Mice

**Figure 5:**
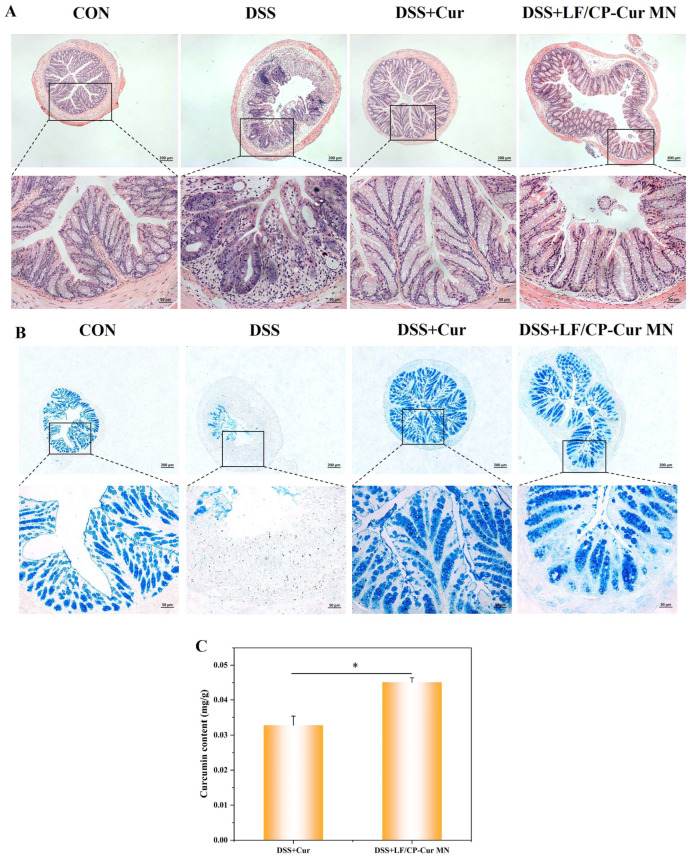
Effect of LF/CP-Cur MN administration on intestinal mucosal barrier. (**A**) H&E staining results, (**B**) AB staining results, (**C**) residual amount of Cur in colon. Data is shown as mean ± SEM (*n* = 3); * *p* < 0.05.

In the DSS-induced IBD model mice, the lamina propria and crypt regions exhibited marked edema accompanied by extensive mononuclear cell infiltration, resulting in a marked disruption of normal tissue architecture. Severe inflammatory damage resulted in multiple erosions on the colonic epithelial surface in IBD mice, accompanied by localized disruption of epithelial layer integrity (Figure 5A). Additionally, significant edema was observed in the submucosal layer. Both free Cur and LF/CP-Cur MN administrations significantly mitigated the DSS-induced colonic tissue edema and mononuclear cell infiltration, suggesting a protective effect mediated through the preservation of epithelial barrier integrity.

Assessment of the colonic mucus barrier by AB staining revealed that normal mice exhibited a dense population of mucus-producing goblet cells within the lamina propria and a well-preserved mucus layer covering the epithelial surface (Figure 5B). This mucus layer consists of an inner, tightly adherent layer that serves as a barrier against bacterial and toxin penetration, and an outer, loose layer that functions as a habitat and nutrient source for commensal bacteria [28]. In contrast, mucus-producing goblet cells were nearly absent in the lamina propria of IBD mice, and the epithelial mucus layer was scarcely detectable. Treatment with either LF/CP-Cur MN or free Cur led to a noticeable recovery in the number of goblet cells and the mucus layer coverage. Given that CP has been shown to promote mucus secretion [29], our results demonstrate that LF/CP-Cur MN counteracts inflammation-driven goblet cell depletion. This nanoparticle enhances the primary physical barrier by stimulating mucus secretion.

The Ca^2+^-induced LF/CP carrier technology significantly improved the colonic delivery efficiency of Cur, as evidenced by a higher residual concentration (0.0451 ± 0.00127 mg/g) compared to free Cur (0.0328 ± 0.00254 mg/g) at 5 h post-administration (Figure 5C). This finding aligns with the well-documented mucoadhesive properties of CP, which contributed to prolonged colonic retention [30]. The enhanced efficiency is presumably via two mechanisms: nano-encapsulation protects Cur from degradation by gastric acid [31]. Abundant carboxyl groups in CP promoted adhesion to the colonic mucus layer through hydrogen bonding and electrostatic interactions with mucins [32]. This ultimately increases the bioaccessibility of Cur.

### 3.6. Effects of LF/CP-Cur MN on the Intestinal Microbiota Structure in IBD Mice

**Figure 6:**
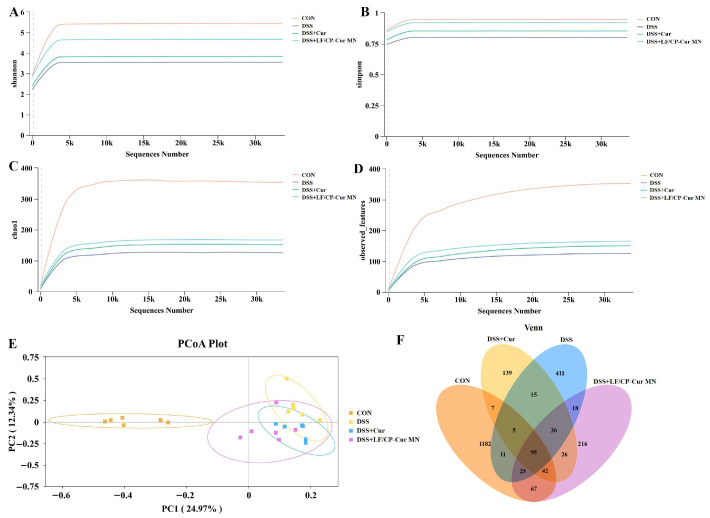
The overall structural presentation of gut microbiota. (**A**) Shannon rarefaction curve, (**B**) Chao1 index, (**C**) OTU numbers, (**D**) Principal component analysis, (**E**) Principal coordinates analysis (PCoA) plot, (**F**) Venn. Data are shown (*n* = 6).

The gut microbiota primarily colonizes the mucus layer on the surface of colonic epithelial cells, and variations in the thickness of this layer play a critical regulatory role in shaping the overall structure of the microbial community [33]. Meanwhile, the diversity of the microbial community within a habitat, as a quantitative attribute of its structure, can be characterized by the number of different organism types and their abundance distribution. This metric has been linked to the onset and progression of IBD [34]. Alpha diversity analysis (Figure 6A–D) in this study revealed that the Chao1 index, observed features, Shannon index, and Simpson index of IBD mice administered with LF/CP-Cur MN were most similar to those of the CON group. In contrast, the DSS and DSS + Cur groups showed notable deviations. Furthermore, beta diversity analysis (principal coordinates analysis (PCoA) plot) (Figure 6E) and Venn diagram analysis (Figure 6F) of shared microbial components demonstrated a greater degree of similarity between the DSS + LF/CP-Cur MN group and the CON group, whereas overlap with the DSS group and the DSS and DSS + Cur group remained limited. These findings indicate that LF/CP-Cur MN is significantly more effective than free Cur in restoring the structural stability of the gut microbiota in IBD mice. This enhanced efficacy is primarily attributed to the LF/CP core–shell structure, which effectively maintains microbial homeostasis, potentially through the ability of CP to increase the relative abundance of beneficial microorganisms.

### 3.7. Role of LF/CP-Cur MN in Maintaining Gut Microbiota Diversity in IBD Mice

The gut microbiota is predominantly composed of strict anaerobes, which exceed facultative anaerobes and aerobic microorganisms in abundance by two to three orders of magnitude. Although the bacterial kingdom encompasses over 50 phyla, the composition of the gut microbiota is predominantly shaped by two major phyla—*Bacteroidetes* and *Firmicutes*. Other phyla are present in relatively minor proportions [35]. These include *Proteobacteria*, *Actinobacteria*, *Deferribacterota*, *Fusobacteria*, and *Cyanobacteria*.

**Figure 7:**
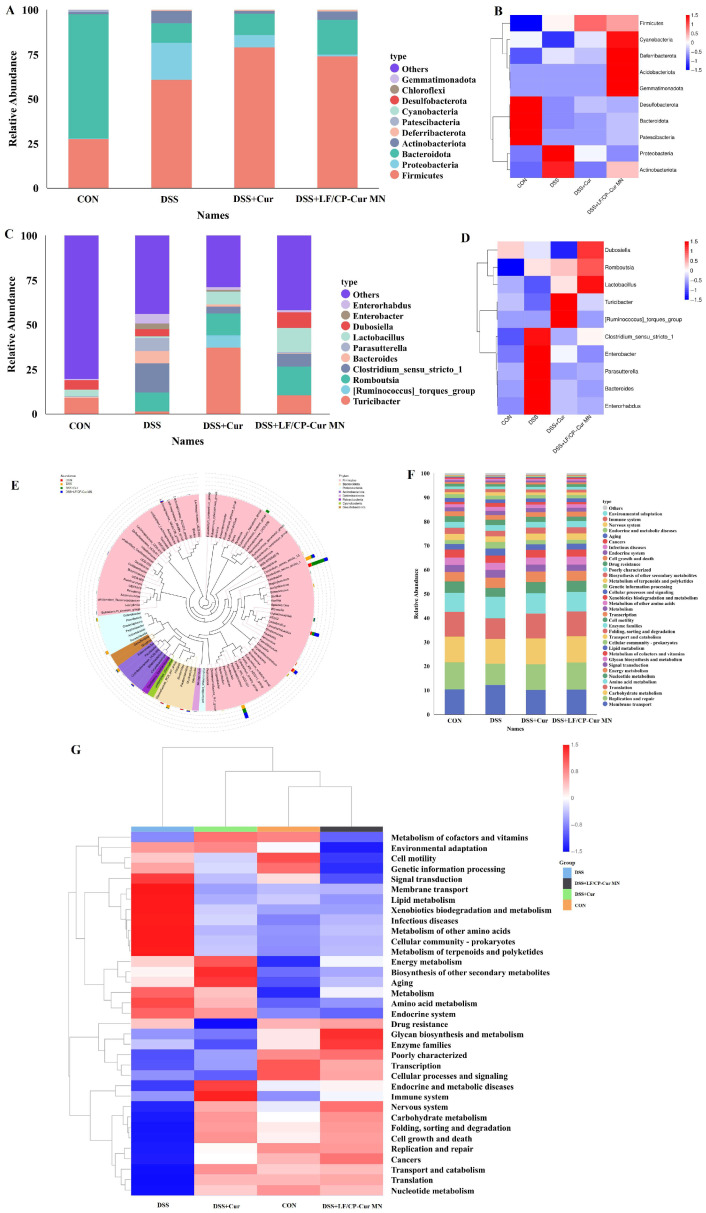
Characterization of gut microbiota composition and associated functions. (**A**) Showing the relative abundance of major microbial phyla via a stacked bar plot. (**B**) Displaying a hierarchical clustering heatmap of phylum-level abundance, where red dots mark significantly altered species upon dietary intervention, with darkness indicating abundance. (**C**,**D**) Presenting genus-level relative abundance and clustering patterns in a stacked bar plot and a heatmap, respectively. (**E**) Depicting the phylogenetic relationships among microbiota at the genus level. (**F**,**G**) Illustrating the relative abundance and clustering of predicted microbial functional profiles. Data are shown (*n* = 6).

The results of this study revealed that, in comparison to the CON mice, the relative abundances of *Bacteroidetes* and *Cyanobacteria* in IBD mice were reduced by 83.93% and 93.95%, respectively. By contrast, the relative abundances of *Firmicutes*, *Proteobacteria*, *Deferribacterota*, *Desulfobacterota*, and *Actinobacteria* exhibited increases of 119.47%, 6094.97%, 5640.00%, and 426.50%, respectively (Figure 7A). Further analysis indicated that after LF/CP-Cur MN intervention, the relative abundances of *Proteobacteria* and *Actinobacteria* in IBD mice decreased by 96.37% and 32.31%, respectively, whereas the relative abundances of *Firmicutes*, *Bacteroidetes*, *Deferribacterota*, and *Cyanobacteria* increased by 21.81%, 75.00%, 194.77%, and 3473.33%, respectively. Notably, compared to the DSS + Cur group, the DSS + LF/CP-Cur MN group showed increased relative abundances of *Bacteroidetes*, *Actinobacteria*, *Deferribacterota*, and *Cyanobacteria* by 66.01%, 162.89%, 257.72%, and 135.09%, respectively, whereas the relative abundances of *Firmicutes*, *Proteobacteria*, and *Desulfobacterota* were reduced by 6.30%, 89.15%, and 44.44%, respectively. The heatmap results demonstrated that LF/CP-Cur MN significantly modulated commensal bacteria such as *Firmicutes*, *Cyanobacteria*, and *Deferribacterota* (Figure 7B), thereby reshaping a healthier microbial community structure and providing mechanistic insight into its therapeutic effects on IBD.

Genus-level analysis revealed a profoundly dysbiotic microbiota in IBD mice, characterized by an overwhelming expansion of *Clostridium_sensu_stricto_1* (increased by 17,069.99%) and the appearance of *Enterobacter*, alongside a collapse of beneficial *Firmicutes* (e.g., *Turicibacter*, *Lactobacillus*) (Figure 7C,D). The LF/CP-Cur MN intervention effectively counteracted this dysbiosis by drastically reducing pathogenic genera, including *Clostridium_sensu_stricto_1*, *Parasutterella*, and *Enterobacter*, with reductions ranging from 57.08% to 98.24%, while remarkably restoring key commensal bacteria such as *Lactobacillus* (increase of 1280.87%) and *Dubosiella*. The superior efficacy of LF/CP-Cur MN compared to free Cur underscores its enhanced ability to selectively inhibit pathogenic microorganisms while promoting the growth of beneficial microbiota, thereby supporting its therapeutic mechanism in IBD. Mao et al. demonstrated that RG-I-enriched pectin significantly increased the abundance of probiotics such as *Bifidobacterium* and *Lactobacillus*, and elevated the levels of SCFAs [36]. LF/CP-Cur MN represents the integration of the biological functionality of Cur and the prebiotic function of pectin into a dual functionality.

Although IBD mice showed an overall increase in the relative abundance of *Firmicutes* at the phylum level, the relative abundances of potentially beneficial genera within this phylum, namely *Lactobacillus* and *Dubosiella*, were significantly reduced. Following LF/CP-Cur MN intervention, the abundances of both genera were markedly restored (Figure 7E). Extensive studies have demonstrated that probiotic strains of *Lactobacillus* promote mucin secretion, enhance the expression of tight junction protein, modulate specific immune pathways, and exert anti-inflammatory effects via the gut–brain and gut–liver axes, thereby contributing to the restoration and maintenance of intestinal barrier integrity in IBD [37,38]. Moreover, recent research has revealed that *Dubosiella* serves as a key producer of SCFAs and plays a critical role in regulating the colonic Treg/Th17 immune balance, thereby attenuating colonic inflammation and promoting the restoration of the mucosal barrier [39]. LF/CP-Cur MN treatment also significantly suppressed the expansion of facultative anaerobes such as *Enterobacter*. It is well established that *Enterobacter* utilizes molybdenum cofactor-dependent anaerobic respiratory enzymes and formate dehydrogenases to confer a growth advantage under inflammation-associated dysbiotic conditions, thereby contributing to the exacerbation of IBD progression [40]. Further functional analysis of the gut microbiome (Figure 7F,G) revealed that membrane transport was enhanced in IBD mice, whereas functions including replication and repair, carbohydrate metabolism, signal transduction, nucleotide metabolism, and metabolism of cofactors and vitamins were attenuated. Both the Cur and LF/CP-Cur MN interventions facilitated the normalization of these functional profiles. In summary, we propose that LF/CP-Cur MN may effectively ameliorate IBD by promoting the growth of beneficial bacteria (e.g., *Lactobacillus* and *Dubosiella*), while suppressing the proliferation of harmful bacteria (e.g., *Enterobacter*), thereby maintaining microbial community structure and improving microbial metabolic function.

### 3.8. SCFAs Elevation by LF/CP-Cur MN in IBD Mice

**Figure 8:**
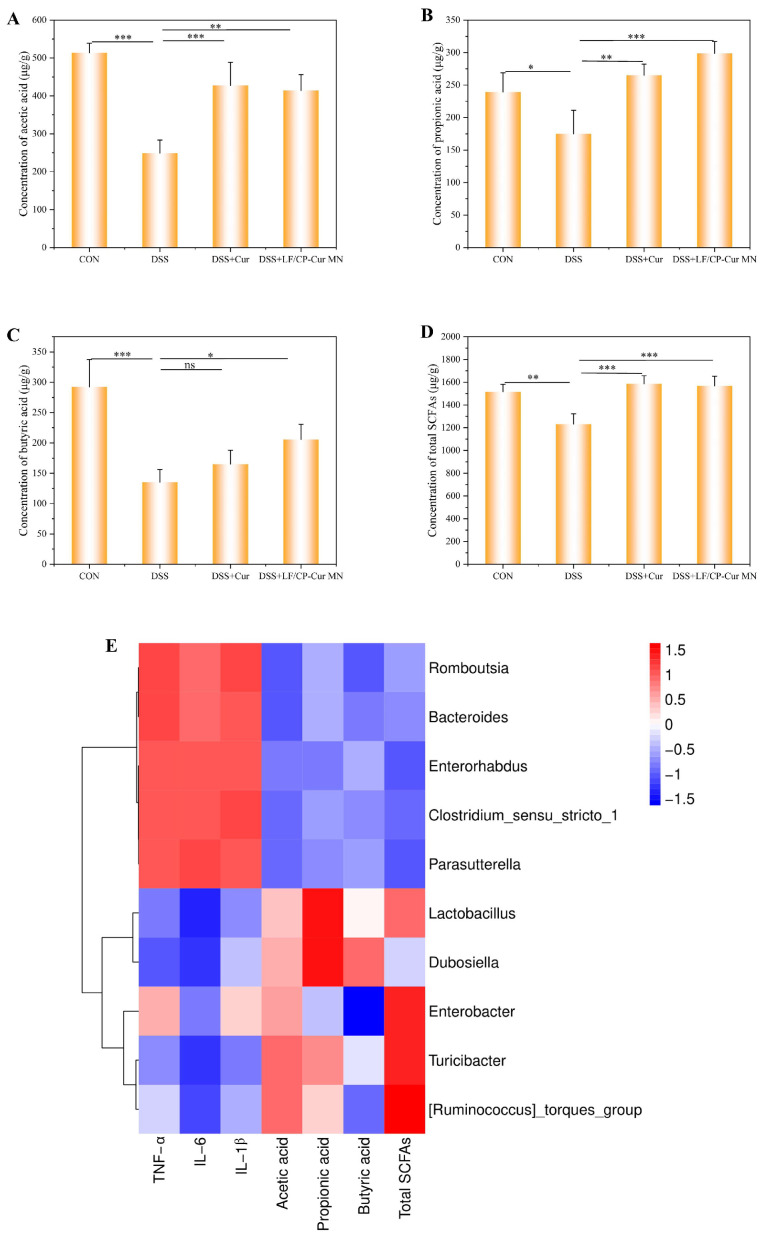
Effect of LF/CP-Cur MN on SCFAs content in colon of mice with IBD. (**A**) Concentration of acetic acid. (**B**) Concentration of propionic acid. (**C**) Concentration of butyric acid. (**D**) Concentration of total SCFAs. (**E**) Correlation analysis. Data showed mean ± SEM (*n* = 3); * *p* < 0.05; ** *p* < 0.01; *** *p* < 0.001; ns: not significant.

The metabolites produced by beneficial gut bacteria, such as SCFAs, can modulate host lipid metabolism, glucose homeostasis, and inflammatory responses through the regulation of RNA expression [41]. Based on this, we conducted a quantitative analysis of SCFAs levels in fecal samples from mice in each experimental group using targeted metabolomics. The results revealed that, in comparison with the CON group, the concentrations of acetic acid, propionic acid, and butyric acid in IBD model mice were significantly reduced by 51.52%, 26.90%, and 53.75%, respectively (Figure 8A–D). After free Cur intervention, the levels of acetic acid, propionic acid, and total SCFAs in IBD mice increased by 71.66%, 51.60%, and 28.91%, respectively. Notably, treatment with LF/CP-Cur MN not only further enhanced the concentrations of acetic acid, propionic acid, and total SCFAs but also significantly elevated butyric acid levels (*p* < 0.05). This effect may be associated with the increased relative abundance of *Lactobacillus* and *Dubosiella* (Figure 8E).

**Figure 9:**
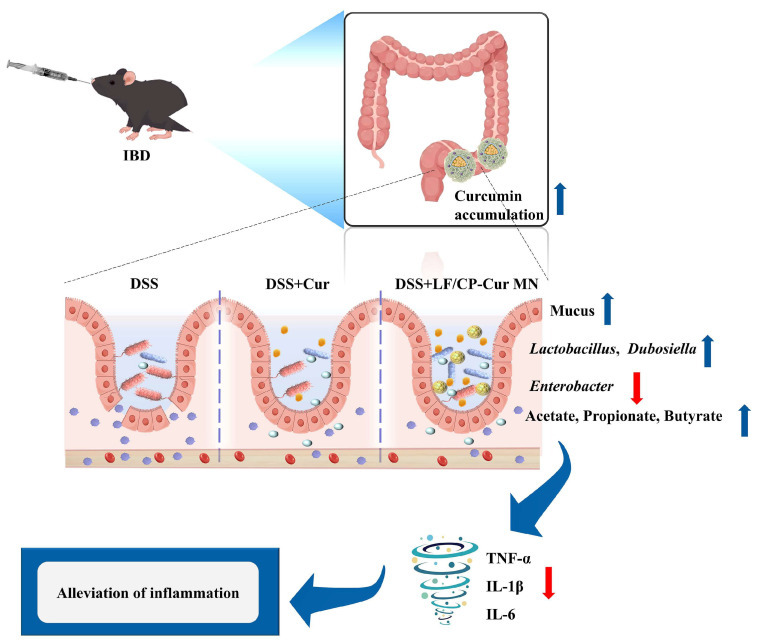
Mechanism of LF/CP-Cur MN in alleviating IBD.

LF/CP-Cur MN modulated the composition of the gut microbiota by promoting the proliferation of beneficial bacteria (such as *Lactobacillus* and *Dubosiella*) while simultaneously reducing the relative abundance of pathogenic taxa, thereby facilitating the production of SCFAs. These SCFAs further stimulated mucus secretion, reinforcing the intestinal mucosal physical barrier (the first line of defense) and improving the microenvironment for intestinal epithelial cells, which in turn promoted the repair of the mucosal barrier. Concurrently, Cur exerted potent anti-inflammatory effects, suppressing inflammatory responses and significantly decreasing the levels of pro-inflammatory cytokines, including TNF-α, IL-6 and IL-1β. In summary, the core–shell structured LF/CP-Cur MN not only preserves the inherent anti-inflammatory activity of Cur but also contributes to maintaining ecological balance through modulation of the gut microbiota, thereby demonstrating a dual therapeutic benefit (Figure 9).

Despite the efficacy of LF/CP-Cur MN in alleviating IBD, certain limitations of this study warrant consideration. On the one hand, although the DSS-induced colitis model is a widely utilized and well-characterized murine surrogate for IBD, it primarily recapitulates the features of acute UC triggered by epithelial barrier disruption. Consequently, the therapeutic effects of LF/CP-Cur MN observed herein should be interpreted within the constraints of this acute injury model. On the other hand, inherent physiological disparities exist between mice and humans regarding gastrointestinal function, including gut microbiota composition, intestinal transit time, and drug metabolism [42]. Notably, the gut microbiota of mice maintained under specific pathogen-free (SPF) conditions differs significantly from that of humans in terms of diversity and dominant taxa. Thus, the microbiota-mediated mechanisms proposed in this study may not be directly extrapolatable to real-world human environments. Future investigations are warranted to further validate the efficacy and safety of LF/CP-Cur MN in chronic models for the management of human IBD.

## 4. Conclusions

This study successfully developed Ca^2+^-induced LF/CP core–shell structured microgel nanoparticles (LF/CP-Cur MN) for the effective encapsulation of hydrophobic Cur. Single-factor optimization established optimal preparation conditions: 1.0 mg/mL CP, 1.5 mg/mL Cur, and 3.0 mg/mL LF. The resulting LF/CP-Cur MN exhibited a loading efficiency of 30.75 ± 2.18%, average particle size of ~208 nm, and zeta potential of ~–32.1 mV. The LF/CP-Cur MN effectively masked the bitterness and astringency of Cur. Furthermore, in vitro release studies demonstrated that the LF/CP-Cur MN exhibited sustained-release properties by delaying the release of Cur. In a DSS-induced IBD murine model, LF/CP-Cur MN effectively alleviated body weight loss, reduced serum levels of the pro-inflammatory cytokines—TNF-α, IL-1β, and IL-6—by 57.19%, 46.58%, and 84.95%, respectively, promoted the repair of the intestinal mucosal barrier through restoration of the epithelial lining and mucus layer, and enhanced the targeted delivery and accumulation of Cur in the colon. Compared to free Cur, the LF/CP-Cur MN promoted the proliferation of beneficial bacteria such as *Lactobacillus* and *Dubosiella*, while inhibiting the growth of *Enterobacteriaceae*, resulting in increased levels of acetate, propionate, butyrate, and total SCFAs. In conclusion, the electrostatic complex-based nano-delivery system formed by LF and CP provides a natural macromolecular platform for the delivery of Cur, enabling a dietary strategy to IBD management through coordinated protection of bioactivity and modulation of the gut microenvironment.

## Data Availability

The original contributions presented in this study are included in the article. Further inquiries can be directed to the corresponding authors.

## References

[1] Lu Q., Yang M.F., Liang Y.J., Xu J., Xu H.M., Nie Y.Q., Wang L.S., Yao J., Li D.F. (2022). Immunology of inflammatory bowel disease: Molecular mechanisms and therapeutics. J. Inflamm. Res..

[2] Kaplan G.G., Ng S.C. (2017). Understanding and preventing the global increase of inflammatory bowel disease. Gastroenterology.

[3] Park K.T., Ehrlich O.G., Allen J.I., Meadows P., Szigethy E.M., Henrichsen K., Kim S.C., Lawton R.C., Murphy S.M., Regueiro M. (2020). The cost of inflammatory bowel disease: An initiative from the Crohn’s & colitis foundation. Inflamm. Bowel Dis..

[4] Ghouri Y.A., Tahan V., Shen B. (2020). Secondary causes of inflammatory bowel diseases. World J. Gastroenterol..

[5] de Boer N.K.H., van Bodegraven A.A., Jharap B., de Graaf P., Mulder C.J.J. (2007). Drug insight: Pharmacology and toxicity of thiopurine therapy in patients with IBD. Nat. Rev. Gastroenterol. Hepatol..

[6] Sandborn W.J., Panés J., D’Haens G.R., Sands B.E., Su C.Y., Moscariello M., Jones T., Pedersen R., Friedman G.S., Lawendy N. (2019). Safety of tofacitinib for treatment of ulcerative colitis, Based on 4.4 years of data from global clinical trials. Clin. Gastroenterol. Hepatol..

[7] Liu F., Li D., Wang X.J., Cui Y., Li X.L. (2020). Polyphenols intervention is an effective strategy to ameliorate inflammatory bowel disease: A systematic review and meta-analysis. Int. J. Food Sci. Nutr..

[8] Martin D.A., Bolling B.W. (2015). A review of the efficacy of dietary polyphenols in experimental models of inflammatory bowel diseases. Food Funct..

[9] Vecchi Brumatti L., Marcuzzi A., Tricarico P.M., Zanin V., Girardelli M., Bianco A.M. (2014). Curcumin and Inflammatory Bowel Disease: Potential and Limits of Innovative Treatments. Molecules.

[10] Farzaneh F., Sarina B., Milad A., Al A., Mohammad Hossein P., Mahmood K.M., Amirhossein S., Hamed M. (2021). Curcumin and inflammatory bowel diseases: From in vitro studies to clinical trials. Mol. Immunol..

[11] Peng Y., Ao M.Y., Dong B.H., Jiang Y.X., Yu L.Y., Chen Z.M., Hu C.J., Xu R.C. (2021). Anti-inflammatory effects of curcumin in the inflammatory diseases: Status, limitations and countermeasures. Drug Des. Dev. Ther..

[12] Liang D.S., Shen X.F., Han L., Ren H., Zang T., Tan L.L., Lu Z.X., Liao X.P., Vetha B.S.S., Liu Y.H. (2024). Dual-ROS sensitive moieties conjugate inhibits curcumin oxidative degradation for colitis precise therapy. Adv. Heal. Mater..

[13] Jin J.M., Ye X.Z., Huang Z.F., Jiang S.C., Lin D.N. (2024). Curcumin@Fe/Tannic acid complex nanoparticles for inflammatory bowel disease treatment. ACS Omega.

[14] Xie Y.Z., Xu W., Jin Z., Zhao K. (2023). Chondroitin sulfate functionalized palmitic acid and cysteine cografted-quaternized chitosan for CD44 and gut microbiota dual-targeted delivery of curcumin. Mater. Today Bio.

[15] Yan J.K., Qiu W.-Y., Wang Y.Y., Wu J.Y. (2017). Biocompatible polyelectrolyte complex nanoparticles from lactoferrin and pectin as potential vehicles for antioxidative curcumin. J. Agric. Food Chem..

[16] Zhou F., Mai T., Wang Z., Zeng Z., Shi J., Zhang F., Kong N., Jiang H., Guo L., Xu M. (2023). The improvement of intestinal dysbiosis and hepatic metabolic dysfunction in dextran sulfate sodium-induced colitis mice: Effects of curcumin. J. Gastroenterol. Hepatol..

[17] Zhao H., Yue Y., Han H., Chen X., Lu Y., Zheng J., Hou H., Lang X., He L., Hu Q. (2017). Effect of toll-like receptor 3 agonist poly I:C on intestinal mucosa and epithelial barrier function in mouse models of acute colitis. World J. Gastroenterol..

[18] Elzoghby A.O., Abdelmoneem M.A., Hassanin I.A., Abd Elwakil M.M., Elnaggar M.A., Mokhtar S., Fang J.Y., Elkhodairy K.A. (2020). Lactoferrin, a multi-functional glycoprotein: Active therapeutic, drug nanocarrier & targeting ligand. Biomaterials.

[19] Lelis C.A., Nunes N.M., Paula H.M.C.D., Coelho Y.L., Silva L.H.M.D., Pires A.C.D.S. (2020). Insights into protein-curcumin interactions: Kinetics and thermodynamics of curcumin and lactoferrin binding. Food Hydrocoll..

[20] Cao L.Q., Lu W., Mata A., Nishinari K., Fang Y.P. (2020). Egg-box model-based gelation of alginate and pectin: A review. Carbohydr. Polym..

[21] Xue J.Y., Luo Y.C. (2023). Protein-polysaccharide nanocomplexes as nanocarriers for delivery of curcumin: A comprehensive review on preparation methods and encapsulation mechanisms. J. Future Foods.

[22] Rahman M., Farooq S. (2025). Role of peanut oleosomes in the delivery of curcumin embedded in interpenetrating emulsion-filled gels made with whey protein and chitosan. Colloids Surf. A.

[23] Bhujbal S.V., Mitra B., Jain U., Gong Y., Agrawal A., Karki S., Taylor L.S., Kumar S., Zhou Q. (2021). Pharmaceutical amorphous solid dispersion: A review of manufacturing strategies. Acta Pharm. Sin. B.

[24] Synytsya A., Čopíková J. (2015). Fourier transform infrared spectroscopy in the study of dietary fibers. Dietary Fiber: Properties, Recovery, and Applications.

[25] Paswan M., Chandel A.K.S., Malek N.I., Dholakiya B.Z. (2024). Preparation of sodium alginate/Cur-PLA hydrogel Beads for curcumin encapsulation. Int. J. Biol. Macromol..

[26] Camilleri M., Linden D.R. (2016). Measurement of gastrointestinal and colonic motor functions in humans and animals. Cell. Mol. Gastroenterol. Hepatol..

[27] Holt P.R., Katz S., Kirshoff R. (2005). Curcumin therapy in inflammatory bowel disease: A pilot study. Dig. Dis. Sci..

[28] Jin M.Y., Li M.Y., Huang R.M., Wu X.Y., Sun Y.M., Xu Z.L. (2021). Structural features and anti-inflammatory properties of pectic polysaccharides: A review. Trends Food Sci. Technol..

[29] Jin M.-Y., Wu X.-Y., Li M.-Y., Li X.-T., Huang R.-M., Sun Y.-M., Xu Z.-L. (2021). Noni (*Morinda citrifolia* L.) fruit polysaccharides regulated IBD mice via targeting gut microbiota: Association of JNK/ERK/NF-κB signaling pathways. J. Agric. Food Chem..

[30] Bigucci F., Luppi B., Monaco L., Cerchiara T., Zecchi V. (2010). Pectin-based microspheres for colon-specific delivery of vancomycin. J. Pharm. Pharmacol..

[31] Nelson K.M., Dahlin J.L., Bisson J., Graham J., Pauli G.F., Walters M.A. (2017). The essential medicinal chemistry of curcumin. J. Med. Chem..

[32] Thirawong N., Nunthanid J., Puttipipatkhachorn S., Sriamornsak P. (2007). Mucoadhesive properties of various pectins on gastrointestinal mucosa: An in vitro evaluation using texture analyzer. Eur. J. Pharm. Biopharm..

[33] Earle K.A., Billings G., Sigal M., Lichtman J.S., Hansson G.C., Elias J.E., Amieva M.R., Huang K.C., Sonnenburg J.L. (2015). Quantitative imaging of gut microbiota spatial organization. Cell Host Microbe.

[34] Qin J., Li R., Raes J., Arumugam M., Burgdorf K.S., Manichanh C., Nielsen T., Pons N., Levenez F., Yamada T. (2010). A human gut microbial gene catalogue established by metagenomic sequencing. Nature.

[35] Sekirov I., Russell S.L., Antunes L.C.M., Finlay B.B. (2010). Gut microbiota in health and disease. Physiol. Rev..

[36] Mao G., Li S., Orfila C., Shen X., Zhou S., Linhardt R.J., Ye X., Chen S. (2019). Depolymerized RG-I-enriched pectin from citrus segment membranes modulates gut microbiota, increases SCFA production, and promotes the growth of *Bifidobacterium* spp., *Lactobacillus* spp. and *Faecalibaculum* spp. Food Funct..

[37] Li C., Peng K., Xiao S., Long Y., Yu Q. (2023). The role of *Lactobacillus* in inflammatory bowel disease: From actualities to prospects. Cell Death Discov..

[38] Sonakshi R., Aditi S. (2022). Gut microbiome and human health: Exploring how the probiotic genus *Lactobacillus* modulate immune responses. Front. Pharmacol..

[39] Zhang Y., Tu S., Ji X., Wu J., Meng J., Gao J., Shao X., Shi S., Wang G., Qiu J. (2024). *Dubosiella newyorkensis* modulates immune tolerance in colitis via the L-lysine-activated AhR-IDO1-Kyn Pathway. Nat. Commun..

[40] Zhu W., Winter M.G., Byndloss M.X., Spiga L., Duerkop B.A., Hughes E.R., Büttner L., de Lima Romão E., Behrendt C.L., Lopez C.A. (2018). Precision editing of the gut microbiota ameliorates colitis. Nature.

[41] Huang Z., Yao Q., Ma S., Zhou J., Wang X., Meng Q., Liu Y., Yu Z., Chen X. (2025). The synergistic role of gut microbiota and RNA in metabolic diseases: Mechanisms and therapeutic insights. Front. Microbiol..

[42] Hugenholtz F., de Vos W.M. (2018). Mouse models for human intestinal microbiota research: A critical evaluation. Cell. Mol. Life Sci..

